# *Salvia officinalis* L. extract and its new food antioxidant formulations induce apoptosis through mitochondrial/caspase pathway in leukemia L1210 cells

**DOI:** 10.2478/intox-2014-0020

**Published:** 2014-12-30

**Authors:** Soňa Jantová, Roman Hudec, Stanislav Sekretár, Juraj Kučerák, Martina Melušová

**Affiliations:** 1 Institute of Biochemistry, Nutrition and Health Protection, Faculty of Chemical and Food Technology, Slovak University of Technology, Bratislava, Slovakia; 2 Institute of Biotechnology and Food Science, Faculty of Chemical and Food, Technology, Slovak University of Technology, Bratislava, Slovakia

**Keywords:** sage, food antioxidants, L1210 cells, apoptosis, caspase

## Abstract

*Salvia officinalis*, L. (Lamiaceae) is one of the most widespread herbal species used in the area of human health and in the food-processing industry. Salvia and its extracts are known to be a rich source of antioxidants. As shown previously, the crude ethanolic extract of salvia (SE) exerts lower anti-oxidative properties in lard compared to the new salvia food formulations No. 1 (SF1; 32% of SE + 68% of the emulsifier Dimodan S-T) and No. 2 (SF2; 32% of SE + 68% of the emulsifier Topcithin 50).

The aim of the present study was to investigate and compare the effects of the SE and its food formulations SF1 and SF2 on the toxicity and/or proliferation of L1210 leukemia cells. We found that SE and both SF1 and SF2 demonstrated different concentration- and time-dependent cytotoxic/antiproliferative cellular effects already within the first 24 h of the treatment. However, SE was nearly 10 times more effective than the new salvia food formulations SF1 and SF2.

We investigated partially also the molecular mechanisms lying behind the action of SE, SF1 and SF2 induced apoptosis in our cell model. We found an apparent involvement of the mitochondrial/caspase-dependent pathway in the described processes. Nevertheless, further investigation is needed before salvia extract and its new antioxidant formulations can be included among the potential food antioxidants with protective properties against cancer.

## Introduction

*Salvia L*., one of the largest genera of the family Lamiaceae (formerly Labiatae), is represented by over 1000 species, organized in five subgenera (Sclarea, Audibertia, Jungia, Leonia, and Salvia) as herbaceous, suffruticose, or shrubby perennial plants. The genus *Salvia* is one of the herbal plant genuses that has been widely used in traditional medicine all around the world (Tayarani-Najarana *et al.*, 2013) due to its diverse biological activities, including antibacterial, spasmolytic, hemostatic, cytotoxic, and anticancer actions and as well as many others. Since ancient times it has been used in the treatment of various disorders, such as tuberculosis, psoriasis, and seborrhoeic eczemas.

Some members of this genus have even an economic importance because of their use as flavoring agents in perfumery and cosmetics. In food and food-processing industry, a major use is in the form of aqueous infusions as sage tea, which is sold legally either as food or medicine (Walch *et al.*, 2011).

Antioxidant properties of compounds isolated from *S. officinalis* were described elsewhere (Cuvelier *et al.*, 1994; Baricevic & Bartol, 2000; Ben *et al.*, 2006). Wu *et al.* (2012) used the oil stability method to evaluate the antioxidant activities of some components. Of these carnosol, rosmanol, epi-rosmanol, isorosmanol, galdosol, and carnosic acid exhibited remarkably strong activity, which was comparable to that of α-tocopherol. Rosmarinic acid and carnosol were the main compounds of all the antioxidant phenolic extracts isolated from *S. officinalis*. Our diet contains fats and oils rich in polyunsaturated fatty acids that easily undergo undesirable oxidative reactions during food processing (heating) or storage (salt addition, freezing). To prevent such quality loss or to retard the effects of oxidation (Angelo *et al.*, 1996), synthetic phenolic antioxidants (butylhydroxytoluene – BHT, butylhydroxyanisole – BHA, *tert*-butylhydroquinone – TBHQ) are commonly used, yet their safety has been questioned (Namiki, 1990; Barlow *et al.*, 1990). Recent research focuses therefore also on polyphenolic antioxidants existing in some spices and herbs like salvia, but also rosemary or thyme (Shahidi & Wanasundara, 1992; Sekretar *et al.*, 2003, 2004 2006; Mariutti *et al.*, 2011; Wu *et al.*, 2012). The increasing interest in replacing synthetic antioxidants by natural compounds (Willcox *et al.*, 2004) of comparable efficacy would be beneficial not only for sustaining the food quality but it might also positively influence the treatment of various human pathologies. Imbalance between reactive oxygen species (ROS) formation and their elimination is involved in conditions like arthritis, cancer, diabetes, cardiovascular diseases, inflammations, neurological disorders, *etc*. (Halliwell, 1996).

Previously we found that the crude ethanolic extract of salvia (SE) exerted antioxidant activity comparable to BHT and its food antioxidant formulations SF1, SF2 containing a mixture of the emulsifier Dimodan S-T or lecithin Topcithin 50 even surpassed the significant antioxidant properties of SE and stabilized fats against oxidation (Sekretar *et al.*, 2003, 2006). In consistence with all the above mentioned facts, we used model murine leukemia L1210 cells to monitor the cytotoxic/antiproliferative activity and apoptosis induction by *Salvia officinalis* L. extract (SE) and its potent food antioxidant formulations SF1 and SF2.

## Material and methods

### Material

The chemicals used in the experiments were purchased from the following suppliers: RPMI medium, fetal calf serum (FCS), antibiotics (penicillin G, streptomycin), proteinase K and RNA-ase from Biocom (Bratislava, Slovakia), trypan blue, dimethyl sulfoxide (DMSO), ethanol, ethidium bromide (EtBr), propidium iodide (PI), ethylenediaminetetraacetic acid (EDTA), Triton X-100, Tris(hydroxymethyl)aminomethane (Tris), agarose, 2,7-dichlorodihydrofluorescein diacetate (DCFH-DA) and 3,3′-dihexyloxacarbocyanine iodide (DiOC6) from Sigma (St Louis, USA). Emulsifier Dimodan S-T (distilled monoacylglycerols) from Danisco A/S (Denmark). Emulsifier Topcithin 50 (50% soya lecithin in soya oil) from Lucas Meyer (Hamburg, Germany). Phosphate-buffered saline (Dulbecco A) (PBSa) from OXOID (Great Britain).

### Preparation of salvia extract and food formulations

*Salvia officinalis* from Fytopharma company (Malacky, Slovakia) was used for the extract preparation. The starting material (dry salvia leaves, 20 g) was over 4 h successively extracted in 500 ml Soxhlet extractor with 95% ethanol. Using a rotary vacuum evaporator, 3.4 g (17% yield) of yellowish powder was obtained and directly used for cytotoxic evaluation as 100% salvia extract (SE) or for the preparation of food formulation SF1 a SF2 (Sekretár *et al.*, 2003, 2004, 2006). Formulation SF1 was prepared by mixing 32% of crude extract (SE) and 68% of the emulsifier Dimodan S-T. Formulation SF2 was prepared by mixing 32% of crude extract (SE) and 68% of the emulsifier Topcithin 50. Crude *Salvia officinalis L.* extract (SE), food antioxidant formulations SF1 and SF2 were dissolved in 100% DMSO. Final concentration of DMSO never exceeded 0.1% (v/v) in either control or treated cells.

### Cell culture

Murine L1210 leukemia cells (obtained from ATCC, Rockville, MD, USA) were grown in RPMI medium supplemented with 10% FCS, 100 U/ml penicillin and 100 µg/ml streptomycin at 37 °C in a 5% (v/v) CO_2_ incubator. All experiments were performed in Petri dishes (Ø 60 mm). The cells were plated at the density of 8 × 10^4^ cells/ml, cell viability was determined by 0.4% (w/v) Trypan blue staining.

### Growth inhibition assay

An inoculum, 8 × 10^4^ cells/ml in the exponential phase of growth, was used. SE, SF1, SF2 were applied in the final concentration range of 0.1–500 µg/ml. The number of cells was determined by direct counting 24, 48 and 72 h after exposure. The cytotoxic effect of the crude extract and food formulations was evaluated as reported previously (Letasiova *et al.*, 2006).

### Cell cycle measurements

Cells (0.5 × 10^6^) treated with SE (18.75, 25.0, 50 µg/ml), SF1 (150, 200, 300 µg/ml) and SF2 (150, 300, 500 µg/ml) in culture medium for 24 h were harvested, washed twice in phosphate-buffered saline (PBS) and exposed to 0.1% (w/v) Triton X-100 in PBS supplemented with RNA-ase (50 µg/ml) for 25 min at 37 °C. Then DNA was stained by PI (50 µg/ml) for 15 min at 4 °C. The samples were analyzed by an EPICS XL flow cytometer (Beckman Coulter Company, CI, USA), equipped with an argon laser operating at 488 nm for excitation of the PI, with the use of software System II provided by the manufacturer (Letasiova *et al.*, 2006).

### Electrophoretic determination of apoptosis

Cells treated with 1–50 µg/ml of SE, 300 and 500 µg/ml of SF1 and 75–500 µg/ml of SF2 for 4, 8, 12, 24, 48 and 72 h were harvested, washed in PBS and lysed in 100 µl of lysis solution (10 mM Tris, 10 mM EDTA, 0.5% (w/v) Triton X-100) supplemented with proteinase K (1 mg/ml). The samples were then incubated at 37 °C for 1 h and heated at 70 °C for 10 min. Following lysis, RNA-se (200 µg/ml) was added and repeated incubation at 37 °C for 1 h followed. The samples were subjected to electrophoresis at 40 V for 3 h in 1.3% (w/v) agarose gel complemented with EtBr (Jantova *et al.*, 2007).

### Caspase 3 activity assay

Cell lysates were prepared and caspase 3 activity was measured according to the manufacturer‘s protocol (CaspACE^™^ Assay System Colorimetric, Promega Corporation). Briefly, 28 µg of total protein was added to the reaction mixtures containing colorimetric substrate peptides specific for caspase 3 (Ac-DEVD-pNA) at 37 °C for at least 2 h and data were collected every 2 h until the signal got stabilized. Absorbance at 405 nm was determined using microplate reader (Humareader, Wiesbaden, SRN) (Jantova *et al.*, 2008). Protein concentration was determined by Lowry's method (Lowry *et al.*, 1951).

### Detection of caspase 8 and caspase 9 activity

Caspase 8 and caspase 9 activities were measured according to the manufacturer‘s protocol (Caspase-Glo^®^ 8/9 Assay, Promega Corporation, USA). Briefly, 100 µl of Caspase-Glo^™^ 8 Reagent (for measuring caspase 8 activity, containing a specific substrate (Z-LETD-aminoluciferin) and 100 µl of Caspase-Glo^™^ 9 Reagent (for measuring caspase 9 activity, containing a specific substrate (Z-LEHD-aminoluciferin), were added to the test tube with 100 µl of cell suspension containing 50000 cells, mixed, and the luminescent signal was measured immediately for 90 min (Jantova *et al.*, 2008).

### Assessment of mitochondrial membrane potential

The changes in mitochondrial membrane potential were determined using DiOC6. Cells were treated with 50 (SE) and 500 (SF1, SF2) µg/ml for 30 min to detect the changes of mitochondrial membrane potential. The cells were harvested, washed and resuspended in PBS and 4 µl of DiOC6 (40 µM) was added. The cells were incubated at 37 °C for 30 min and analyzed by fluorescence microscopy (Repicky *et al.*, 2009).

### Statistical analysis

Data are presented as means of at least three independent experiments ± SD. Student′s test was used to determine statistically significant differences between the respective mean values and significant changes are highlighted (n.s. not significant; *p<*0.05).

## Results

**Figure 1:**
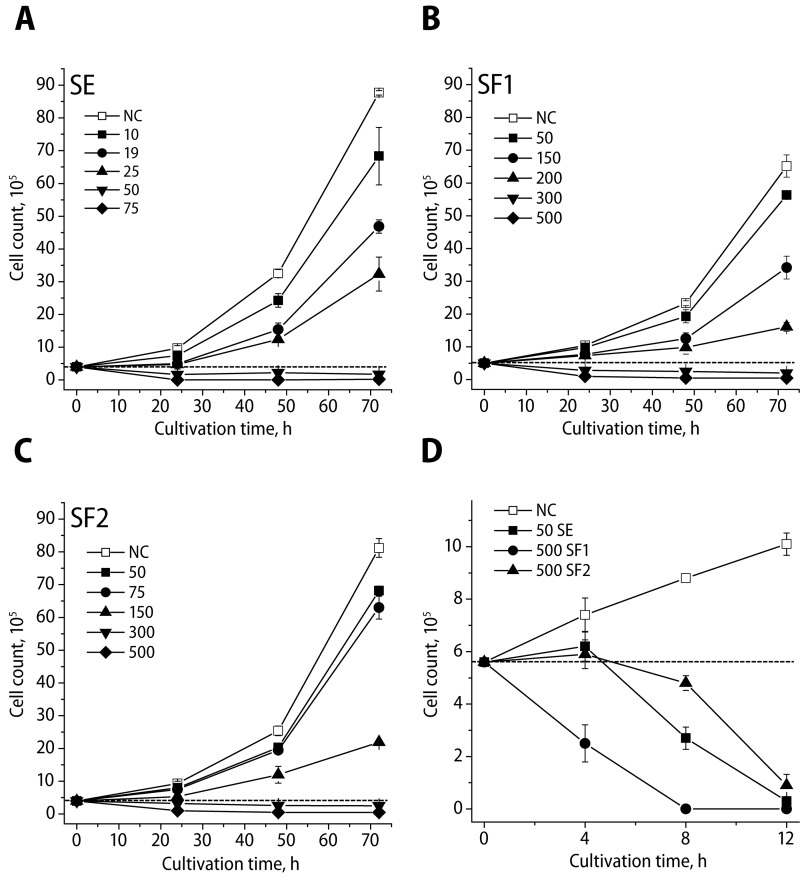
Anti-proliferative effects of the sage extract SE (A) and its formulations SF1 (B), SF2 (C) on L1210 cell. Cells were treated with indicated concentrations for 24, 48 and 72 h and counted. Cytotoxic effect of 50 µg/ml SE and 500 µg/ml SF1 and SF2 on L1210 cells treated for 4, 8 and 12 h (D). Eeach data point represent mean values ± SD of minimum three independent experiments. Application of 0.1% DMSO served as negative control, NC. Dotted lines correspond to cell number value at the beginning of treatment. All concentrations are displayed in µg/ml.

We evaluated the growth inhibition of L1210 cells treated with SE, SF1 and SF2 in concentrations of 0.1–500 µg/ml for 24–72 h (Figure 1). As follows from the growth curves of L1210 cells (Figure 1A, B, C), the highest used concentrations (50 and 75 µg/ml for SE, 300 and 500 µg/ml for SF1 and SF2, resp.) induced an acute cytotoxic effect manifested by immediate decrease of viable L1210 cells after 24 h of exposure. This effect increased in the following time intervals. An intensive antiproliferative activity was demonstrated also by SE concentrations of 18.75 and 25.0 µg/ml and SF1 and SF2 concentrations of 200 and 250 µg/ml. The cytotoxic effect at the other concentrations tested was directly proportional to the concentration and time of influence.

Table 1 shows the values of L1210 growth inhibitory concentrations IC_50_ and IC_100_ of SE, SF1 and SF2 for 24, 48 and 72 h. The values increased with the time of treatment of L1210 cells. The effectivity was highest by using the crude extract (SE), followed by the food antioxidant formulations SF2 and SF1.

**Table 1:** Concentrations of sage extract (SE) and its formulations SF1 and SF2 able to inhibit growth of L1210 cell to 50 (IC_50_) or 100% (IC_100_) upon treatment for 24, 48 and 72 h.

	IC_50_, µg/ml			IC_100_, µg/ml		
	24 h	48 h	72 h	24 h	48 h	72 h
**SE**	12.0±0.8	16.5±1.1	19.3±1.3	19.3±1.3	28.0±1.5	46.8±3.1
**SF1**	124.3±4.1	143.1±5.8	180.8±6.7	103.1±7.7	113.9±6.9	121.6±8.1
**SF2**	263.6±7.4	269.1±8.5	282.5±9.3	223.3±10.4	239.2±11.4	263.8±13.6

Concentrations displayed in µg/ml were calculated from the toxicity curves.

As mentioned above, we observed that the highest concentrations used, *i.e.* 50 and 75 µg/ml for SE and 300 and 500 µg/ml for SF1 and SF2, respectively, caused massive cytotoxicity already within the first 24 h of treatment. Therefore, we evaluated the L1210 cell proliferation within a shorter treatment period with cells counted every 4 h within the final 12 h treatment (Figure 1D). SE, SF1 and SF2 demonstrated distinct time-dependent cytotoxic/antiproliferative effects. Four hours of cell treatment with the crude extract SE and the antioxidant formulation SF2 was sufficient to induce a cytostatic effect only in comparison to the effect of the antioxidant formulation SF1, which within the same treatment time caused already degradation of the treated cell. Finally, prolonged time of cell incubation with all salvia preparations tested caused further cell count decreases below the inoculum cell level, reaching almost complete cell clearance following 12 h of treatment.

To understand the mechanism of cell proliferation inhibition by SE, SF1 and SF2, we next studied their ability to modulate the cell cycle and induction of apoptotic cell death in L1210 cells.

**Figure 2:**
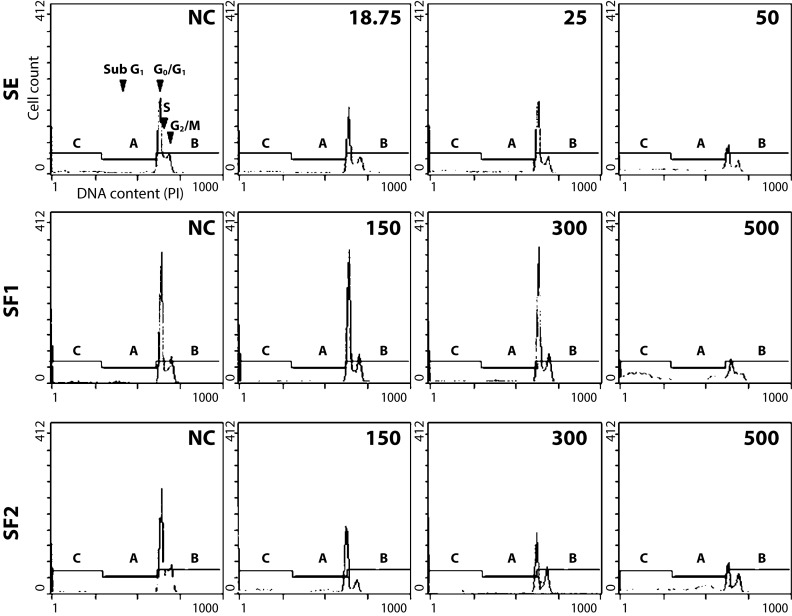
Flow cytometry analysis of cell cycle and apoptosis induction in L1210 cells treated with indicated concentrations of the sage extract (SE) and its formulations SF1 and SF2 for 24 h. Representative flow cytometry histogram are shown. Application of 0.1% DMSO served as negative control, NC. Concentrations are displayed in µg/ml. Cell cycle phases (G_0/_ G_1_, S and G_2_/M) as well as apoptotic sub-population (Sub G_1_) are indicated by arrowheads.

To study the impact on the cell cycle profile, we monitored the effect of 18.75, 25.0 and 50.0 µg/ml of SE; 150, 200 and 300 µg/ml of SF1 and 150, 300 and 500 µg/ml of SF2 in L1210 cells for 24 h (Figure 2). On the one hand, flow cytometry revealed that SE and its formulations did not have any significant effect on the cell cycle profile of L1210 cells. On the other hand, a significant sub-G_0_ fraction was detected in L1210 cells treated for 24 h with concentrations of 50 (SE), 300 (SF1) and 500 (SF2) µg/ml.

**Figure 3:**
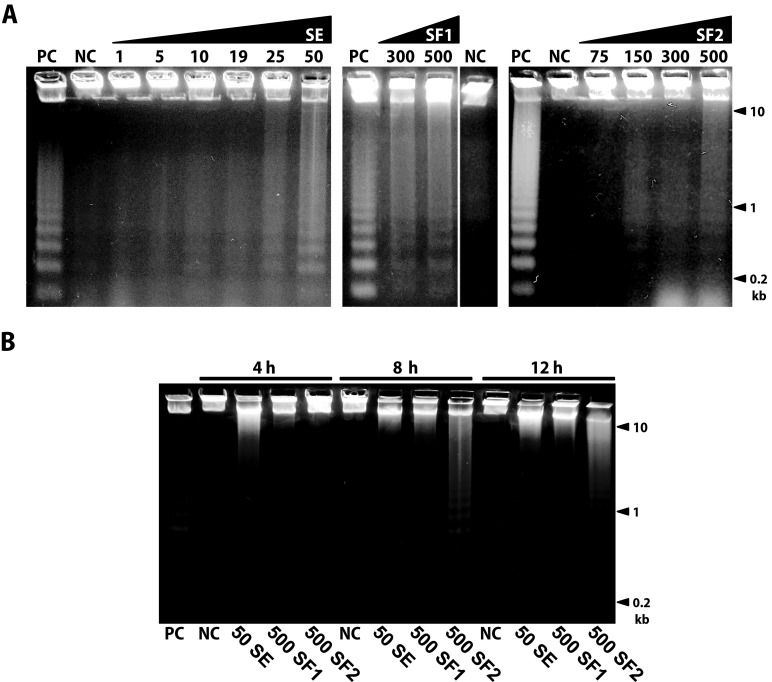
Detection of the apoptotic DNA fragmentation in L1210 cells treated with indicated concentrations of the sage extract (SE) and its formulations SF1 and SF2 for 24 h (A). Cells treated with the SE and SF1, SF2 in concentration 50 and 500 µg/ml, respectively, were cultured for indicated time (B). All concentrations are displayed in µg/ml. Application of 0.1% DMSO served as negative control, NC. Positive control, PC, refers to cells treated with ciplatine as described in Materials and methods. Positions of molecular weight markers are indicated.

Cells treated with concentrations of 50 (SE) and 500 (SF1 and SF2) µg/ml for 4, 8, 12, 24, 48 and 72 h were subjected to agarose gel electrophoresis to assess apoptotic DNA fragmentation. As indicated in Figure 3A, clear apoptotic DNA fragmentation was observed in L1210 cells treated for 24 h with 25 and 50 µg/ml of SE, 300 and 500 µg/ml of SF1 and 150–500 µg/ml of SF2. DNA fragmentation was also found in cells treated for 48 and 78 h with concentrations of 50 µg/ml of SE, 300 and 500 µg/ml of SF1 and 300 and 500 µg/ml of SF2 (data not shown). Furthermore, as indicated in Figure 3B, apoptotic DNA fragmentation was observed in L1210 cells treated with the highest concentrations tested (50 µg/ml of SE, 500 µg/ml of SF1 and SF2) already after 4, 8 as well as 12 h of treatment.

**Figure 4:**
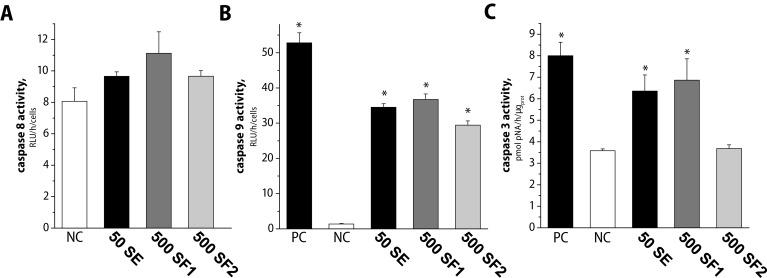
Activity of caspase 8 (A), caspase 9 (B) and caspase 3 (C) were measured in cells treated for 1 and 2 hours, respectively. Sage extract (SE) and formulations SF1, SF2 in concentration 50 and 500 µg/ml were used. Application of 0.1% DMSO served as negative control, NC. Positive control, PC, refers to cells treated with ciplatine as described in Materials and methods. The results are expressed as the mean ± SD of three independent experiments. *p<0.005 compared with NC.

The evidence based on the observation of the apoptotic DNA fragmentation induction in L1210 cells treated with SE, SF1 and SF2 led us to the examine the activities of caspase 8 and 9 as well as caspase 3 (Figure 4). Since activation of caspases 8 and 9 precede activation of executive caspase 3, we measured first their activity. One hour of treatment of the L1210 cells with 50 µg/ml SE and 500 µg/ml SF1 and SF2, was sufficient to induce significant activation of caspase 9 (Figure 4B). Caspase 9 activities upon treatment reached almost 60% activity of caspase 9 induced by 180 µg/ml of cisplatine, used as positive control. Under identical conditions, the activity of caspase 8 was measured and the obtained values were undistinguishable from that measured in control (Figure 4A). Measurement of caspase 3 revealed that 1-h treatment of L1210 cells with 50 µg/ml SE and 500 µg/ml SF1 was sufficient to substantially induce its activity, which was comparable to that of positive control. The activity of caspase 3 in L1210 cells treated with 500 µg/ml SF2 was only slightly increased compared to solvent treated cells (negative control, NC).

**Figure 5:**
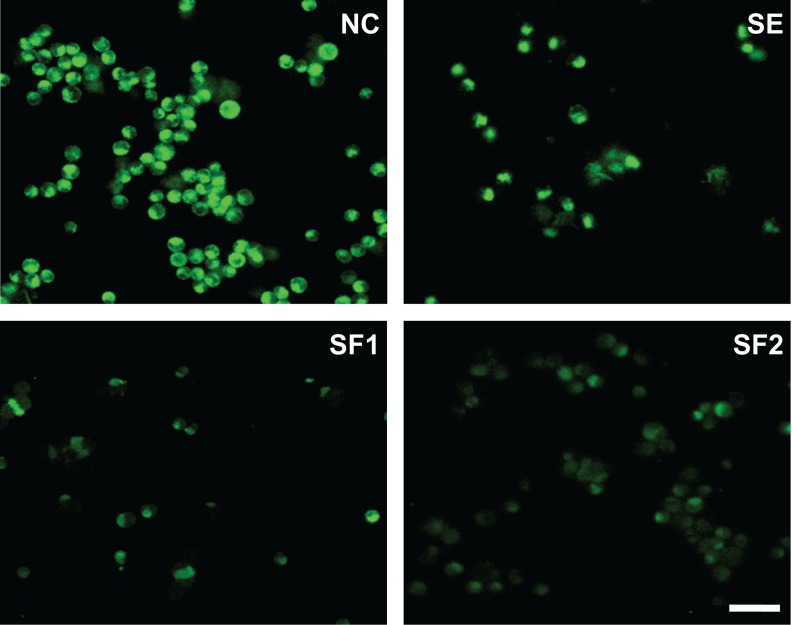
*In situ* fluorescent microscopy of the mitochondrial membrane potential changes induced in L1210 cells upon treatment with the sage extract (SE) and formulations SF1, SF2 in concentration 50 and 500 µg/ml, respectively. Application of 0.1% DMSO served as negative control, NC. Scale bar is 50µm.

Mitochondrial membrane potential decay is one of the accompanying processes of the intrinsic apoptotic pathway activation. To evaluate whether it changed upon treatment of L1210 cells with 50 µg/ml SE and 500 µg/ml SF1 and SF2, we monitored it by using fluorescence sensitive probe DiOC6. As shown in Figure 5, 30-min treatment with SE as well as with SF1 and SF2 caused considerable decrease of the mitochondrial membrane potential in comparison to the solvent treated control (NC).

## Discussion

*Salvia* is a rich source of phytochemicals including flavonoids, sesquiterpenoides, diterpenoides, sesterterpenes and triterpenes. Several active components such as rosmarinic acid, royleanone, horminone, and acetyl horminone have been isolated from the roots of *S. officinalis.* These compounds, extracts and essential oils resulted in human colon, breast, leukemia and lung tumor reduction *in vitro* and reduction in oncogene transformed cells (Liu *et al.*, 2000; Shoemaker *et al.*, 2005; Rozalski *et al.*, 2006; Fiore *et al.,* 2006; Loizzo *et al.*, 2007; Li *et al.,* 2008; Tayarani-Najarana *et al.*, 2013). Some authors also demonstrated protective properties of the genus *Salvia*. Gali-Muhtasib & Affara (2000) reported that the oil extract of the sage plant showed potent suppressive activities against tumor promotion in mouse skin and thus could be an effective chemopreventive agent against skin cancer. Vujosevic and Blagojevic (2004) reported antimutagenic properties of terpenoid fractions of *Salvia officinalis* in the mammalian system *in vivo*. Li *et al.* (2008) found that tanshinone IIA – an antioxidant constituent of *Salvia mitiorrhiza,* could serve as a protective agent in cancer prevention treatment. Celik and Isik (2008) determined the chemopreventive role of *Salvia officinalis* infusion on antioxidative defense systems in rats.

Natural antioxidants may act not only as direct ROS scavengers but they might also modulate enzymes involved in ROS elimination, drug metabolism or repair responses as well as working as signaling molecules in important cascades for cell survival (Lima *et al.*, 2005, 2007a b). On balance, identification and study of novel substances from natural sources possessing antioxidant activities are important strategies to improve human health condition and life quality.

With the aim to obtain new natural antioxidants to enhance the stability of fats and oils with anticancer properties, we prepared a crude ethanolic extract from dry leaves of *Salvia officinalis* (SE) and food antioxidant formulations SF1 and SF2 containing 32% of SE with respective addition of emulsifiers Dimodan S-T and Topcithin 50 (Sekretar *et al.*, 2003, 2004 and 2006). Previously we found that the antioxidant activity of our salvia extract was comparable to BHT, a synthetic phenolic antioxidant commonly used in fats and oils to retard oxidation. The crude extract contains dyes or heavy metals, which accelerate oxidation and so the extract is not directly suitable as food antioxidant. We concluded that safe food formulations prepared in this manner manifested significant antioxidant properties and stabilized fats against their oxidation (Sekretar *et al.*, 2003). Krajcova *et al.* (2008) tested the effects of this extract and its formulations for their activity against common microbial contaminants of food and cosmetics products (*Bacillus cereus*, *Escherichia coli*, *Pseudomonas aeruginosa*, *Staphylococcus aureus*, *Listeria monocytogenes*). The authors confirmed the hypothesis that the substances acted in food not only as antioxidants but also as antimicrobial agents.

In the present study, we evaluated the cytotoxic/antiproliferative activity of salvia crude extract (SE) and its food antioxidant formulations SF1 and SF2 on murine leukemia L1210 cells. As follows from the growth curves of the L1210 cells (Figure 1), salvia extract and its formulations caused different cytotoxic effects depending on the concentration, time of exposure and substance composition. The highest used concentrations of the crude extract (SE) and its food formulations (SF1 and SF2) induced an acute cytotoxic effect manifested by immediate cell degeneration and lysis. This cytolytic effect was increased with prolonged time of treatment and it was proportional to the concentration used and the time of exposure.

Cytotoxic activity of *Salvia officinalis* extract on leukemia cells was reported by Shahneh *et al.* (2013a, b). The authors found that methanolic extract caused dose dependent inhibition of cell proliferation of human leukemic monocyte cell line U937 and human acute myelocytic leukemia cell line KG-1A with IC_50_ values 205.11 µg/ml (for U937 cells) and 179.00 µg/ml(for KG-1A cells) after 24 h of treatment. As seen in Tab. 1, our IC_50_ values obtained on murine leukemia cell line L1210 were lower than on U937 and KG-1A cells. It is thus plausible to conclude that the murine leukemia L1210 cells were more sensitive than human leukemia cells U937 and KG-1A to *Salvia officinalis L.* extract.

The addition of emulsifiers to the crude extract to create salvia food formulations, decreases the cytotoxicity compared to crude salvia extract (Figure 1). It is surprising, since Sekretar *et al.* (2003, 2006) found that these food formulations (SF1 and SF2) exhibited greater antioxidant potency compared to crude salvia extract (SE). This lower cytotoxicity might however be due to decreased salvia content in food formulations compared to 100% salvia extract.

The cell cycle analysis by flow cytometry (Figure 2) showed that neither SE nor its food antioxidant formulations SF1 a SF2 changed significantly the cell cycle profile of treated L1210 cells. What we observed was that the highest used concentration (50 µg/ml for SE, 500 µg/ml for SF1 and SF2) caused accumulation of cells in the sub G_1_ population, which indicates induction of apoptotic cell death. This assumption was confirmed by the detection of intranucleosomal DNA fragmentation (Figure 3). The DNA fragmentation was detectable already after 4 h of treatment with all substances used in their highest concentrations tested (Figure 3B).

The ability of salvia extracts or salvia components to induce apoptosis of different cell lines was reported by many authors (Li *et al.*, 2000; Liu *et al.*, 2000; Oh *et al.*, 2002; Park *et al.*, 2007). Apoptotic cell death of leukemia cells induced by the methanolic extract of *Salvia officinalis* L. in the concentration range 100–300 µg/ml was demonstrated by Shahneh *et al.* (2013a, b). The authors reported that the extract evoked dose-dependent induction of apoptosis of human leukemia cells U937 and KG-1A after 24 h of treatment. In comparison to our results, we conclude that lower concentrations of SE and SF1 and SF2 as well as shorter time of exposure were needed to induce apoptosis in murine leukemia L1210 cells.

To reveal the molecular mechanism involved in SE, SF1 and SF2-induced apoptosis in L1210 cells, we studied their effects on caspase 9, 8 and 3 and mitochondrial membrane potential. As shown in Figure 4, cells treated with SE, SF1, SF2 have activated caspases 9 and subsequently caspase 3, while the mitochondrial membrane potential is decreased (Figure 5). Caspase 8 activation was not observed, which may indicate that all substances tested induced apoptosis only by induction of the intrinsic mitochondrial pathway.

In summary, on the basis of the obtained results we can conclude that SE, SF1 and SF2 demonstrated different concentration- and time-dependent cytotoxic/antiproliferative effects on murine leukemia L1210 cells. The cytotoxicity of the substances tested was already detectable within the first 24 h of treatment. Crude salvia extract (SE) as well as its food formulations SF1 and SF2 induced apoptosis of L1210 cells by activation of caspases 9 and 3, as well as by mitochondrial membrane potential decay. On balance, the results clearly show that SE, SF1 and SF2 induce apoptosis of L1210 cells through the mitochondrial/caspase-dependent pathway.

Nature is a limitless source of substances that may replace synthetic ones to serve the purpose of protection against food alterations and its nutritional and quality degradation. *Salvia officinalis* L. can be one of such natural sources and our ethanolic extract SE and/or the new food antioxidant formulations SF1 and SF2 can be used to increase the shelf life of food, and moreover, they may serve as potential anti-leukemia agents.
